# Cholera in New Orleans

**Published:** 1849-04

**Authors:** 


### CHOLERA IN NEW ORLEANS.

Dr. Hester, editor of the New Orleans Medical and Surgical Journal, states the following facts in regard to cholera as it existed in that city.

The disease, as is well understood, broke out about the time that the ship Swanton arrived at New Orleans from Havre, but the cases occurred in different parts of the city, and remote from this vessel.

The disease was chiefly confined to the intemperate, the reckless, and such as were exposed, badly fed, lodged, and clothed; the blacks, as falling under this class, were great sufferers.

Old residents were not so subject to attacks as strangers newly arrived in the city.

Dr. H. has not heard of a single case of cholera among the citizens who passed through the epidemic in 1832. The disease was particularly fatal to persons affected with chronic diarrhea.

Influenza prevailed to some extent with cholera, and Dr. H. remarked that individuals who were affected with the former escaped the latter complaint.

Dr. H. says, in conclusion, "congestion kills the patient, therefore let us regard it as an acute form of *algide*—of *congestive* fever, and nothing more."

